# Polar flagella rotation in *Vibrio parahaemolyticus* confers resistance to bacteriophage infection

**DOI:** 10.1038/srep26147

**Published:** 2016-05-18

**Authors:** Hui Zhang, Lu Li, Zhe Zhao, Daxin Peng, Xiaohui Zhou

**Affiliations:** 1 Jiangsu Key Laboratory of Food Quality and Safety-State Key Laboratory Cultivation Base of MOST, Jiangsu Academy of Agricultural Sciences, Nanjing 210014, China; 2 Department of Pathobiology & Veterinary Science, The University of Connecticut, 61 N. Eagleville Road, Storrs, CT 06269-3089, USA; 3 Center of Excellence for Vaccine Research, The University of Connecticut, 61 N. Eagleville Road, Storrs, CT 06269-3089, USA; 4 Key Laboratory of Tropical Marine Bio-resources and Ecology, Guangdong Provincial Key Laboratory of Applied Marine Biology, South China Sea Institute of Oceanology, Chinese Academy of Sciences, Guangzhou, China; 5 College of Veterinary Medicine, Yangzhou University, Yangzhou, Jiangsu, PR China

## Abstract

Bacteriophage has been recognized as a novel approach to treat bacterial infectious diseases. However, phage resistance may reduce the efficacy of phage therapy. Here, we described a mechanism of bacterial resistance to phage infections. In Gram-negative enteric pathogen *Vibrio parahaemolyticus*, we found that polar flagella can reduce the phage infectivity. Deletion of polar flagella, but not the lateral flagella, can dramatically promote the adsorption of phage to the bacteria and enhances the phage infectivity to *V. parahaemolyticus*, indicating that polar flagella play an inhibitory role in the phage infection. Notably, it is the rotation, not the physical presence, of polar flagella that inhibits the phage infection of *V. parahaemolyticus*. Strikingly, phage dramatically reduces the virulence of *V. parahaemolyticus* only when polar flagella were absent both *in vitro* and *in vivo*. These results indicated that polar flagella rotation is a previously unidentified mechanism that confers bacteriophage resistance.

*Vibrio parahaemolyticus* is the leading cause of diarrhea linked to the consumption of contaminated seafood worldwide^1^. Currently, the most common treatment for *V. parahaemolyticus* infection is antibiotics. However, increasing prevalence of antimicrobial resistance in *V. parahaemolyticus*, presumably due to the extensive use of antimicrobials in clinical treatment and aquaculture systems, was recently reported^2^,^3^,^4^. For instance, recent studies have shown that all isolates of *V. parahaemolyticus* are resistant to ampicillin and cephazolin and 50% of the clinical and environmental isolates are multi-drug resistant^2^,^3^,^4^. Particularly, recent isolates of *V. parahaemolyticus* have been shown to be resistant to new front-line antibiotics, e.g., fluoroquinolones and extended-spectrum cephalosporins^5^. Emergence of *Vibrio* species that are resistant to multiple antibiotics is a serious global problem and suggests that alternative treatment and prevention strategies are needed.

Lytic bacteriophages (phages) that are widespread in nature are a group of viruses that can invade various bacterial species and eventually lyse the bacterial cells^6^. Studies have demonstrated that phages have the potential to alleviate infectious diseases caused by various bacterial pathogens^7^. One of the key advantages for phage therapy is that phages are active against antibiotic-resistant bacteria and they usually do not disturb beneficial microbiota^7^,^8^. The initial step for phages to invade their hosts is adsorption^9^. Adsorption is one the most intricate steps for the entire lytic cycle because phages must recognize specific bacterial components^10^. The primary receptors that are recognized by phages include bacterial surface-located proteins (e.g., outer membrane protein)^11^,^12^,^13^,^14^, lipopolysaccharide^15^ and teichoic acids^16^. Resistance to phage adsorption occurs when these receptors are altered or masked by extracellular matrix or other structures^17^.

Recent studies have shown that lateral flagella are required for adsorption of phages to some bacterial species, e.g., *Salmonella enterica* serovar Typhimurium and *Agrobacterium. sp*^18^,^19^. These flagellatropic phages actively interact with flagella to increase the concentration of phage particles around the receptors, thus facilitating the phage-receptor interactions and consequent phage adsorption. Some *Vibrio* species, e.g., *V. parahaemolyticus* and *V. alginolyticus* contain two distinct types of flagella system: polar flagella and lateral flagella. Polar flagellum is located on the cell pole and is required for bacterial swimming in soft agar, while lateral flagella is responsible for bacterial swarming in solid agar^20^,^21^. Although phages that can infect *V. parahaemolyticus* have been isolated and some of them exhibited therapeutic efficacy for the diseases caused by *V. parahaemolytics*^22^,^23^,^24^, the role of each flagella system in phage infection is unknown.

In this study, we first isolated a new *V. parahaemolyticus* phage (phage OWB) and determined the role of polar and lateral flagella in phage infection. Our results demonstrated that polar flagella can reduce the phage adsorption by blocking phage attachment to the bacterial cells. In contrast, lateral flagella had a minimal role in phage infection. Further analysis showed that it is the rotation, not the physical presence, of polar flagella that reduces the phage infectivity. Phage OWB significantly reduced the cytotoxicity of polar flagella mutant, but not WT, against HeLa cells. In animal model, phage OWB dramatically reduced the colonization of polar and lateral flagella mutant in the small intestine of infant rabbits. These results demonstrated that polar flagella rotation is a previously unidentified mechanism that confers bacteriophage resistance in *V. parahaemolyticus*.

## Results

### Isolation and characterization of a new *V. parahaemolyticus* phage

**Figure 1:**
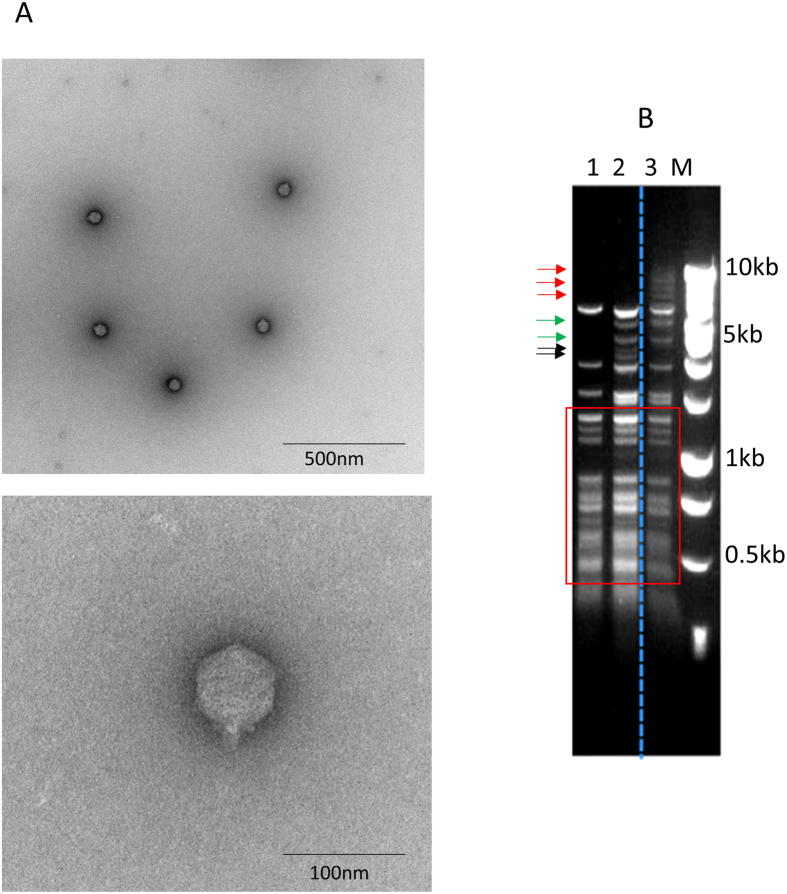
Characterization of a bacteriophage OWB that infects *parahaemolyticus.* Electron microscopy showed that phage OWB had a short tail and isometric capsid ((**A**), upper panel). High magnification of the phage was also shown ((**A**), lower panel) Restriction digestion indicated that phage OWB (lane 3) had different genome content compared with two phages described previously (VPMS1 in lane 1 and VpaM in lane 2) (**B**). The bands in the red boxes are the same among the three phages. Bands pointed by red arrows are present in phage OWB, but are absent in the other two phages. Bands pointed by green arrows are present in phage OWB and VpaM, but are absent in VPMS1. Bands pointed by black arrows are present in VpaM, but are absent in VPMS1 and OWB (**B**). Bar = 500 nm for the electron micrograph.

We collected sea water sample from the Atlantic Ocean and used *V. parahaemolyticus* strain RMID 2210633^25^, a clinical strain that harbors both polar and lateral flagella, to isolate bacteriophage that can infect *V. parahaemolyticus*. A new phage vB_VpaS_OWB (designated as OWB thereafter) was isolated. Electron microscopy showed that OWB had a short tail of 13 nm and a 65-nm isometric capsid (Fig. 1A). According to the International Committee on Taxonomy of Viruses (ICTV), OWB was classified into Podoviridae family. To determine if phage OWB is similar to previously sequenced *V. parahaemolyticus* phages: VPMS1^24^ and VpaM^23^, we isolated genomic DNA from phage OWB, VPMS1 and VpaM and digested DNA with restriction enzyme HhaI. In the restriction profile, OWB had three distinct bands (with the size between 7 and 10 KB) that are not present in both VPMS1 and VpaM (Fig. 1B, lane 3, red arrows). Compared to VPMS1 (Fig. 1B, lane 1), OWB also had two extra bands (Fig. 1B, lane 3, green arrows). Furthermore, two bands that are present in VpaM (Fig. 1B, lane 2, black arrows) are absent in OWB (Fig. 1B, lane 3). These result indicated that phage OWB is different from these two sequenced phages.

### Phage OWB inhibits the growth of *V. parahaemolyticus* lacking polar flagella

**Figure 2:**
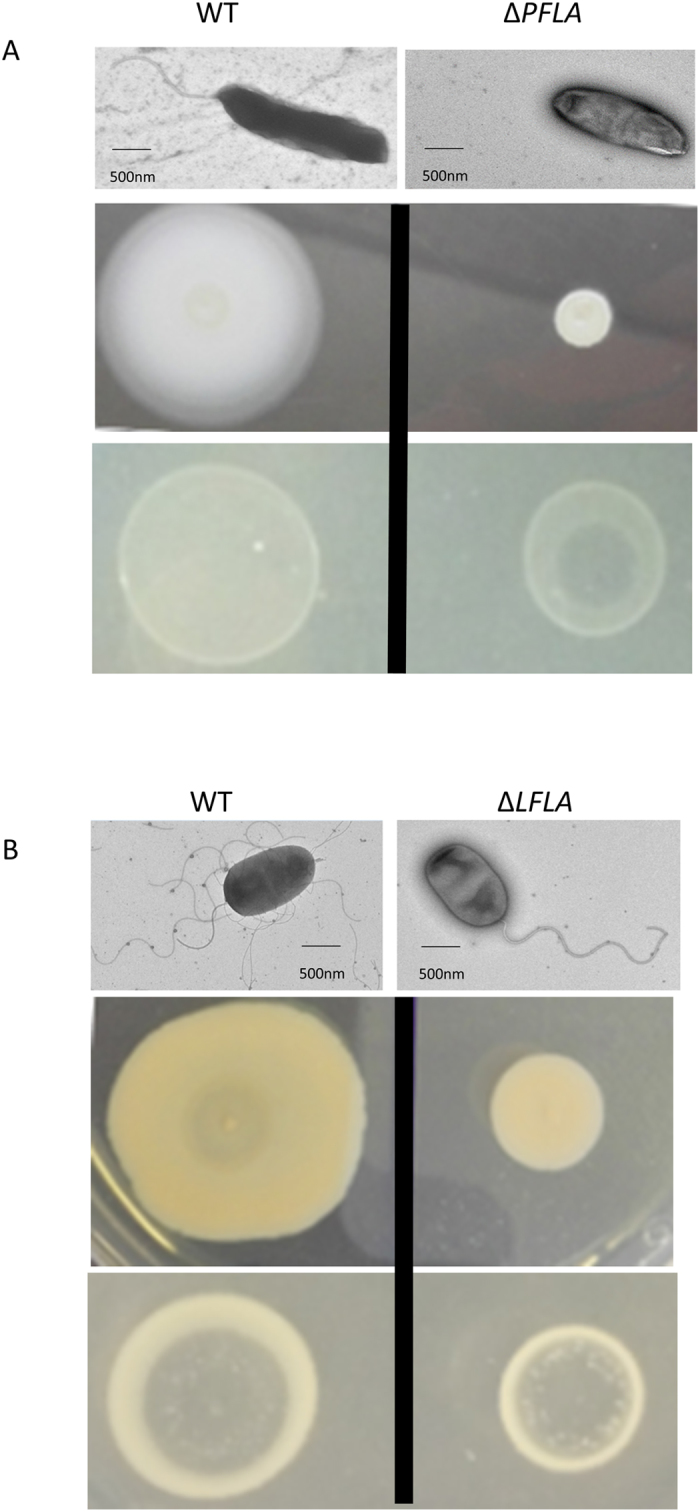
Characterization of *V. parahaemolyticus* flagella, motility and phage sensitivity. (**A**) Polar flagella is present in WT type but not in the mutant that lacks genes encoding the rod of polar flagella (∆*PFLA*) when bacteria were grown in the swimming plate (LB medium containing 1.0% NaCl and 0.3% agar) (upper panel). Swimming ability of ∆*PFLA* was reduced compared to that of WT (middle panel). On the swimming plate, WT is resistant to the phage infection, while ∆*PFLA* is sensitive to phage infection (lower panel). (**B**) Lateral flagella is present in WT type but not in the mutant that lacks genes encoding the rod of lateral flagella (∆*PFLA*) when bacteria were grown in the swarm plate (brain heart infusion medium containing 1.5% Bacto agar and 1% NaCl) (upper panel). Swarm ability of ∆*LFLA* was reduced compared to that of WT (middle panel). On the swarm plate, both WT and ∆*LFLA* are sensitive to phage infection.

**Figure 3:**
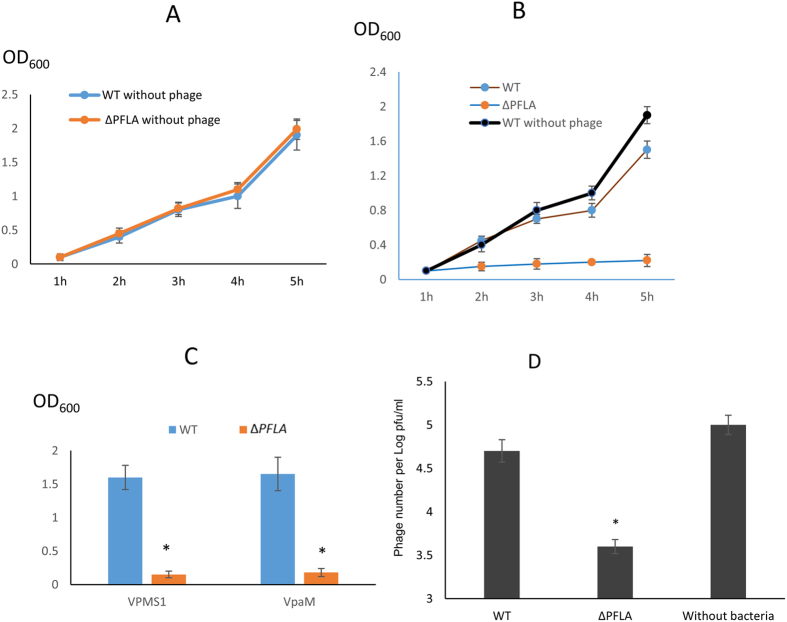
Phage OWB inhibits the growth of *V. parahaemolyticus* lacking polar flagella. (**A**) Growth curve of *V. parahaemolyticus* WT and flagella mutants in the absence of phage OWB in LB medium. Each experiment was repeated for three times and representative data were presented. (**B**) Growth curve of *V. parahaemolyticus* WT and ∆*PFLA* in the presence of phage OWB. The growth curve of a WT strain in the absence of phage OWB was included as a control (black solid cycle). (**C**) OD_600_ was measured for *V. parahaemolyticus* culture after 5 h of growth in the presence of phage VPMS1 and VpaM. Each experiment was repeated for three times and average data with standard deviation were reported. Stars (^*^) indicates statistical difference (*P* < 0.05) compared to the WT. (**D**) Phage adsorption was illustrated as free phages that are not adsorbed to *V. parahaemolyticus*. Equal pfu of phage was added to the culture of *V. parahaemolyticus*, and following 1 h of adsorption, culture was centrifuged and the free phage in the supernatant was determined. Each experiment was repeated for three times and average data with standard deviation were reported. Stars (^*^) indicates statistical difference (*P* < 0.05) compared to the WT.

It has been previously reported that peritrichous (or lateral) flagella could promote the attachment of phage to bacteria and thus enhance the phage infectivity in *Salmonella* and *Agrobacterium*^18^,^19^. *V. parahaemolyticus* contains two types of flagella system: polar flagella and lateral flagella. We wanted to determine if polar and lateral flagella play different roles in phage infection. We created *V. parahaemolyticus* mutant strains that lack polar flagella or lateral flagella (Table 1). As expected, deletion of polar flagella (∆*PFLA*) significantly reduced the bacterial swimming ability on soft agar (0.3%) (Fig. 2A, middle panel), while deletion of lateral flagella (∆*LFLA*) abolished swarming ability on solid agar (1.5%) (Fig. 2B, middle panel). Electron microscopy analysis further showed that, on the soft agar, WT only produces polar flagella, while ∆*PFLA* does not produce any flagella (Fig. 2A, upper panel), verifying that polar flagella on the soft agar is functional. Further electron microscopy analysis showed that, on the swarm plate, WT produces lateral flagella, while ∆*PFLA* does not produce lateral flagella, indicating that lateral flagella is functional on the swarm plate. It is worth noting that mutation of lateral flagella does not affect the production of polar flagella (Fig. 2B, upper panel) under the conditions favoring bacterial swarm. To determine the role of polar flagella on phage infectivity, we performed a phage drop assay by placing a drop of phage on top of the bacterial culture spot on the soft agar. A clear zone at the center of the spot would indicate inhibition of bacterial growth. Following 5 h of growth, a clear zone was observed at the center of ∆*PFLA*, while the clear zones were not observed at the center of WT (Fig. 2A, lower panel). These results indicated that mutation of polar flagella enhanced the phage infectivity to *V. parahaemolyticus*. To determine the role of lateral flagella on phage infectivity, we performed phage drop assay on the swarm plate under which condition lateral flagella are functional (Fig. 2B, upper and middle panels). Clear zone was observed at the center of both WT and ∆*LFLA* strains (Fig. 2B, lower panel), indicating that lateral flagella do not play a significant role in phage infection. We then performed growth curve of WT and ∆*PFLA* in the presence or absence of phage. We did not include the ∆*LFLA* strain because lateral flagella were not produced in the liquid medium. In the absence of phage, ∆*PFLA* and WT *V. parahaemolyticus* had similar growth curve (Fig. 3A). In the presence of phage, growth of ∆*PFLA* was dramatically inhibited (*P* < 0.05 at 2, 3, 4 and 5 h after inoculation compared to the growth of ∆*PFLA* in the absence of phage) (Fig. 3B), while the growth of WT was only slightly inhibited (*P* < 0.05 only at 5 h after inoculation compared to the growth of WT in the absence of phage) (Fig. 3B). Growth of ∆*PFLA*, but not WT, was also dramatically inhibited by the two sequenced phages VPMS1 and VpaM (Fig. 3C). These results indicated that polar flagella can reduce the infectivity of bacteriophage.

### Polar flagella reduce the adsorption of the phage to *V. parahaemolyticus*

**Figure 4:**
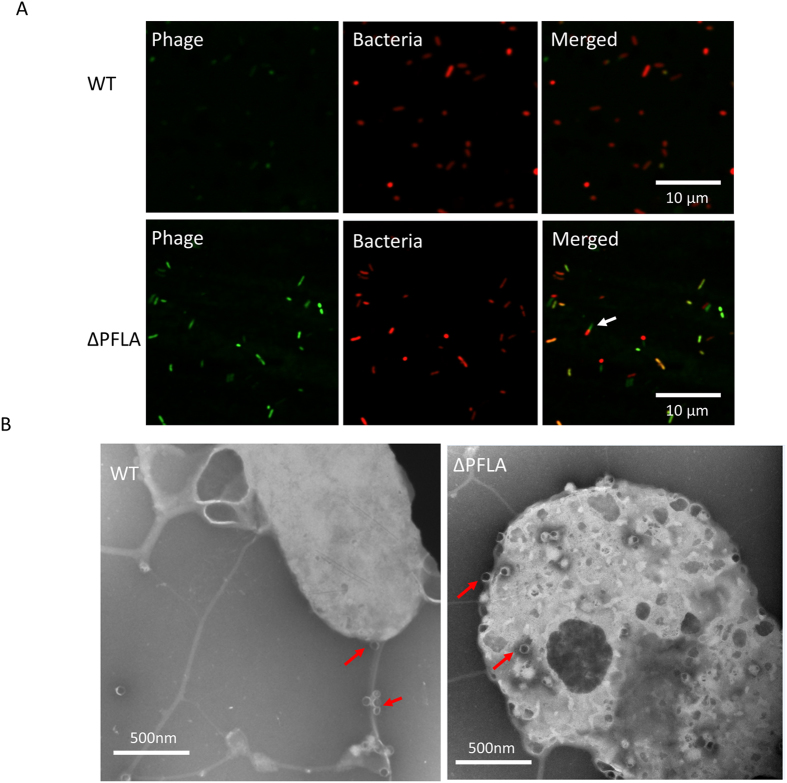
Polar flagella inhibits the attachment of phage OWB to *V. parahaemolyticus*. (**A**) SYBR green-labeled phage OWB was incubated with *V. parahaemolyticus* containing a red fluorescence protein plasmid for 1 h and imaged by a fluorescence microscope. White arrows indicate the attachment of SYBR green-labeled phage OWB to the pole of *V. parahaemolyticus*. (**B**) *V. parahaemolyticus* was incubated with phage OWB (MOI = 10) for 2 hours and processed for electron microscopy analysis. Red arrows indicate phage particles.

We explored how polar flagella affect phage infectivity. As adsorption is the first step of phage infection, we determined if polar flagella affect adsorption. Adsorption of phage OWB to *V. parahaemolyticus* was determined by measuring free phage plaque forming unit (pfu) in the medium after incubation of equal pfu of phage with WT and ∆*PFLA*. The results showed that there were significantly higher pfu of free phage in the medium after incubation with WT than that after incubation with ∆*PFLA* (*P* < 0.05) (Fig. 3D). These results indicated that polar flagella reduce the adsorption of phage to *V. parahaemolyticus*. To further characterize the role of polar flagella in the adsorption of phage OWB to *V. parahaemolyticus*, we stained phage OWB with SYBR green. The stained phage OWB was used to infect WT and ∆*PFLA* that express red fluorescence protein RFP from a plasmid. Fluorescent microscopy analysis showed that green fluorescence was present when ∆*PFLA* was incubated with phage OWB, while green fluorescence was very weak when WT was incubated with phage OWB (Fig. 4A). These results further indicate that polar flagella inhibit the attachment of OWB to *V. parahaemolyticus*. It is worth noting that some of the green fluorescence was present at the pole of *V. parahaemolyticus* ∆*PFLA* (Fig. 4A, arrow), while the majority of the green fluorescence was present throughout the bacterial surface. Further electron microscopy analysis showed that significantly more phages were present on the surface of ∆*PFLA* than those present on WT (Fig. 4B). These results suggest that that phage OWB initiates the attachment to *V. parahaemolyticus* and such attachment was reduced by the polar flagella. ∆*PFLA* that was infected with phage OWB was swelling and blebing and became slightly sphere, while WT that was infected with phage OWB exhibited normal rod shape (Fig. 4B), indicating that phage OWB was lytic when polar flagella was absent. Notably, when the polar flagella are present, some phages that attach to the polar flagella are visible (Fig. 4B, arrows).

### Rotation of polar flagella contributes to the decreased phage infectivity

**Figure 5:**
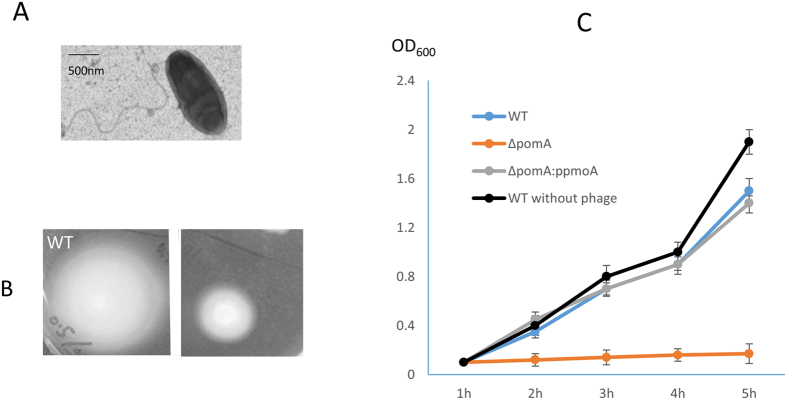
Rotation of polar flagella reduces the phage infectivity. (**A**) Mutation of *pomA* does not affect the polar flagella production. (**B**) Mutation of *pomA* reduces bacterial swimming ability. (**C**) Growth curve of WT, ∆*pomA*, ∆*pomA*: p*pomA* in the presence of phage OWB (**C**). The growth curve of WT in the absence of phage was included as a control (**C**, black solid circle).

We further determined the underlying mechanisms by which polar flagella reduce phage infectivity. We made a mutant of *V. parahaemolyticus* by deleting the entire open reading frame of *pomA*, a gene that is required for the rotation, but not for the production and assembly of polar flagella. As expected, polar flagella were produced at the pole of ∆*pomA* (Fig. 5A); however, the swimming ability of ∆*pomA* was reduced (Fig. 5B), indicating that polar flagella in ∆*pomA* is not functional, although they are produced and assembled. Growth curve analysis showed that the growth of ∆*pomA* was significantly reduced by phage OWB, while the complemented strain had similar growth curve with WT in the presence of phage (Fig. 5C). These results indicated that rotation of polar flagella, not the physical presence of polar flagella, can reduce the phage infectivity.

### Phage OWB reduces the cytotoxicity of *V. parahaemolyticus* against HeLa cells when polar flagella are absent

**Figure 6:**
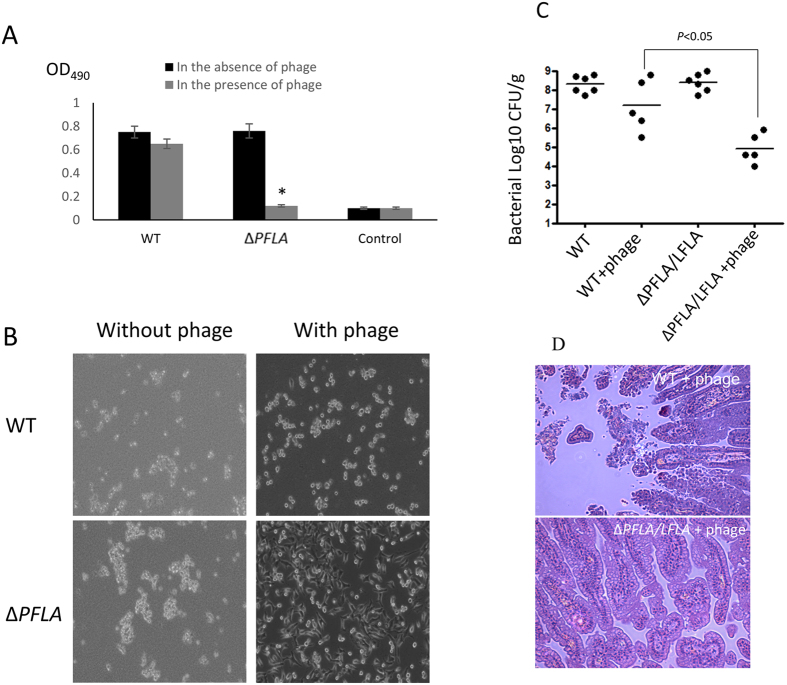
Phage OWB reduces the virulence of *V. parahaemolyticus*. In the absence of phage, WT and polar flagella mutant *V. parahaemolyticus* were equally cytotoxic to HeLa cells (**A**, black bars). In the presence of phage, cytotoxicity of polar flagella mutant was reduced by 90%, while the cytotoxicity of WT was only reduced by 10% compared to those in the absence of phage (**A**). Control indicates no bacteria inoculation (**A**). Stars (^*^) indicates statistical difference (*P* < 0.05) compared to the WT in the presence of phage (**A**). Morphology of HeLa cells inoculated with both phage and *V. parahaemolyticus* (**B**, right panel). Morphology of HeLa cells inoculated *V. parahaemolyticus* alone (**B**, left panel). Colonization of *V. parahaemolyticus* in the small intestine of infant rabbits (CFU/g) with or without phage OWB (**C**). Lines in (**C**) show geometric means based on at least five rabbits. Stars (^*^) indicates statistical difference (*P* < 0.05) compared to the WT. HE staining of intestinal tissues obtained from infant rabbits challenged with both *V. parahaemolyticus* (WT: upper panel; ∆*PFLA*/*LFLA*: lower panel) and phage OWB (**D**).

WT *V. parahaemolyticus* is highly cytotoxic to HeLa cells^26^. Since phage can inhibit the growth of *V. parahaemolyticus* lacking polar flagella, we hypothesized that the cytotoxicity of polar flagella mutant against HeLa cells would be reduced in the presence of phage. To test this hypothesis, WT or ∆*PFLA* was mixed with phage OWB and the mixture was immediately used to inoculate HeLa cells. Cytotoxicity was measured after 5 h of infection. As a control, each strain was used to inoculate HeLa cells in the absence of phage. WT and ∆*PFLA* were equally cytotoxic to HeLa cells in the absence of phage (Fig. 6A, black bars). In the presence of phage, the cytotoxicity of WT against HeLa cells was reduced by 10%, while the cytotoxicity of ∆*PFLA* was reduced by 90% comparing to that in the absence of phage (Fig. 6A, comparison between the black bars and grey bars). The cytotoxicity of ∆*PFLA* was significantly lower than that of WT in the presence of phage (Fig. 6A). In the absence of phage, HeLa cells inoculated with WT or ∆*PFLA* all became round and detached from the wells after 5 h of infection (Fig. 6B, left panel), indicating that both WT and flagella mutants were highly cytotoxic to HeLa cells. In the presence of phage, HeLa cells inoculated with ∆*PFLA* strains exhibited normal morphology and still firmly attached to the wells. In contrast, HeLa cells inoculated with WT became round and detached from the well (Fig. 6B, right panel). These results indicated that phage OWB reduces the cytotoxicity of *V. parahaemolyticus* against HeLa cells when polar flagella were absent.

### Phage OWB reduces intestinal colonization of *V. parahaemolyticus* lacking polar flagella

WT *V. parahaemolyticus* colonizes the small intestine and cause severe diarrhea disease following oral inoculation of the infant rabbits^27^. To determine the therapeutic effect of phage OWB, we inoculated infant rabbits with WT and ∆*PFLA*/*LFLA* in the presence or absence phage OWB. In the absence of phage OWB, both WT and ∆*PFLA*/*LFLA* colonized small intestine at high level (~10^9^ CFU/gram tissue) after 38 h of oral inoculation (Fig. 6C). In the presence of phage OWB, colonization of WT was reduced by 10-fold, while colonization of ∆*PFLA*/*LFLA* was reduced by 1000-fold comparing to the colonization of these strains in the absence of phage (Fig. 6C). Furthermore, in the presence of phage OWB, colonization of ∆*PFLA*/*LFLA* was significantly lower (100-fold) than that of WT (*P* < 0.05) (Fig. 6C). Histology analysis showed epithelial sloughing and disruption of normal villi in the small intestine of rabbits inoculated with the mixture of WT and phage OWB (Fig. 6D, upper panel). In contrast, epithelial structure was normal in the rabbits inoculated with the mixture of ∆*PFLA*/*LFLA* and phage OWB (Fig. 6D, lower panel). In the absence of phage, both WT and ∆*PFLA*/*LFLA* cause epithelial sloughing and disruption of normal villi in the small intestine of infant rabbits^27^,^28^. These results further demonstrated that phage therapeutic efficacy for the diseases caused by *V. parahaemolyticus* was significantly enhanced when polar flagella were absent.

## Discussion

In this study, we showed that that rotation of polar flagella is the restriction factor for maximum phage infection of *V. parahaemolyticus*. This conclusion was supported by the results that (1) deletion of polar flagella greatly enhanced the ability of phage to lyse *V. parahaemolyticus* (Figs 2 and 3); (2) elimination of polar phage rotation, but not the physical presence of polar flagella also enhance phage’s ability to lyse bacteria (Fig. 5); and (3) therapeutic efficacy of phage for *V. parahaemolyticus* is dramatically enhanced when the polar flagella are mutated (Fig. 6). These results not only provide a phage resistance mechanism, but also set the foundation to develop innovative phage therapy for diseases caused by *V. parahaemolyticus* and potentially other *Vibrio* species.

Bacteriophages are widely distributed in the soil, animal intestine or other environment that are populated by bacterial host. The phage genome is highly diversified, reflecting a high evolution rate when facing antiphage barriers. Comparison of the DNA profile using restriction digestion showed that phage OWB isolated in this study is different from those that were isolated previously (Fig. 1B), consistent with the idea that phage genome is less conserved and highly evolved^6^. Nevertheless, the polar flagella can reduce the infectivity of all the three phages (Fig. 3B,C), indicating that polar flagella-mediated reduction of phage infectivity is not phage specific.

Because of the lytic property, phage has been increasingly recognized as a new approach to develop therapies for bacterial infectious diseases^29^. Phage therapy has the advantage of killing antibiotic resistant bacterial pathogens without affecting beneficial flora, thus representing a new approach to treat bacterial infections^7^,^30^. However, bacteria frequently develop strategies to resist phage infection by preventing phage adsorption and DNA entry and cutting DNA after its entry^17^,^31^. Different bacterial species use distinct strategies to resist phage infections. For example, *Staphylococcus aureus* can produce protein A that masks the phage receptors and thereby reduces phage adsorption^32^. Bacteria can also produce phage resistance by producing exopolysaccharide (EPS) that spatially interferes the interaction between phage and the phage receptors. For phages that use EPS as their receptors, bacteria can modify EPS and modified EPS is no longer recognizable by phages. Better understanding of the phage resistant mechanisms for each particular bacterial species is critical for the development of effective phage therapies against such pathogens.

Flagellum is a long helical structure extruding from the bacterial surface. Some bacterial species, e.g., *Salmonella enterica* serovar Typhimurium only produces one type of flagella, lateral flagella. *Vibrio* species, e.g., *V. parahaemolyticus*^33^ and *V. alginolyticus*^34^, can not only produce lateral flagella, but also polar flagella that are present exclusively on the pole of the bacterial surface. Studies have shown that some phages use lateral flagella to mediate their attachment to the receptors. For example, phage iEPS5 requires the counterclockwise (CCW) rotation of the flagella to infect *Salmonella enterica* serovar Typhimurium, while phage Chi requires the polyhook of the flagella to infect *Salmonella*^18^. Interestingly, in this study, we showed that polar flagella can in fact reduce the adsorption of phage to *V. parahaemolyticus* because deletion of the polar flagella dramatically enhanced the adsorption of phage to *V. parahaemolyticus* (Figs 3 and 4). Such a dramatic difference could be due to the structural and functional difference between polar and lateral flagella^20^,^21^. Polar flagella in *V. parahaemolyticus* are typically covered with a thick sheath, while lateral flagella do not have a sheath. Although both polar and lateral flagella are driven by rotary motors, the source of the power is different. Polar flagella use Na^+^-motive force (SMF), while lateral flagella use H^+^-motive force (PMF) to drive the rotation^35^,^36^. Lateral flagella in *Salmonella* or *E. coli* have a rotation speed of approximately 10,000 rpm, while the polar flagella in *V. alginolyticus* rotate at a speed of approximately 100,000 rpm and propel the bacteria in liquid as fast as 60 μm/s^20^,^37^. Our analysis using fluorescence-labeled phage showed that phage attachment to *V. parahaemolyticus* can be dramatically enhanced if the polar flagella are deleted (Fig. 4), suggesting that polar flagella inhibit the phage attachment to *V. parahaemolyticus*. It is possible that high speed rotation would throw the phages off the polar flagella after initial attachment and thus the chances for the adsorption to the phage receptors are significantly reduced. Alternatively, polar flagella may spatially interfere with phage binding to their receptors, leading to the low level of phage adsorption. Spatial interference with phage attachment has been observed for bacterial cell surface structures. For example, the K1 capsule of *E. coli* directly interferes with phage T7 attachment to its LPS receptor^38^, and thus the receptors are hidden and inaccessible to the phages^39^. Spatial interference can also be due to the production of bacterial decoys. Shedding of outer membrane vesicles (OMVs) into the environment prevents phage adsorption^40^. To differentiate these two possibilities, we generated a *pomA* (PomA is the stator subunit of the polar system) mutant strain in which the polar flagella are still assembled, but the rotation of the polar flagella is abolished^20^,^41^,^42^. Our results showed that abolishing the rotation of polar flagella is sufficient to enhance the phage infectivity (Fig. 5), thus favoring the hypothesis that rotation, but not the spatial interference, reduces the phage adsorption and infectivity. These results are in fact consistent with the phage drop assay on swarm plate where ∆*LFLA* strain produces polar flagella, but lacks the ability to inhibit phage infection (Fig. 2B). Rotation of polar flagella in *V. parahaemolyticus* have been shown to serve as a mechanosensor^43^. Therefore, we do not exclude the possibility that deletion of stator subunit of *V. parahaemolyticus* polar flagella may alter the bacterial surface properties (e.g., EPS production and the expression of phage receptors), which facilitates the phage attachment. It is worth to note that phage OWB was isolated from the sea water sample using the WT RMID 2210633 strain that harbors both polar and lateral flagella. This indicates that although rotation of polar flagella reduces the phage adsorption, it does not completely eliminate the adsorption to *V. parahaemolyticus*.

Phages have been approved to use in food industry to reduce bacterial contamination, while phage-based therapeutics are still facing many challenges^44^,^45^. One of such challenges is the intrinsic bacterial resistance to phage, which would greatly reduce the effectiveness of phage therapy. Our results showed that phage therapeutic efficacy can be significantly enhanced after the polar flagella are deleted from *V. parahaemolyticus*. Combination of bacteriophages with molecules that can inhibit the synthesis or the rotation of polar flagella may represent an effective approach for phage therapy against *V. parahaemolyticus*. High-throughput screening has identified a group of small chemical compound (quinazoline-2,4-diamino analogs) that can suppress the polar flagella assembly and motility in *V. cholera*^46^. Future studies will be directed to determine if combination of such chemical compounds with phages would enhance the effectiveness of phage therapy against diseases caused by *V. cholera* or *V. parahaemolyticus*.

In conclusion, we showed in this study that polar flagella greatly reduce the phage attachment to *V. parahaemolyticus*. Further analysis using *pomA* mutant demonstrated that it is the rotation, not the physical presence, of polar flagella that reduces the phage adsorption to the bacterial cells. Both cell culture and infant rabbit studies have demonstrated that mutation of polar flagella can greatly enhance the effectiveness of phage therapy against *V. parahaemolyticus*.

## Materials and Methods

### Bacteria strains and growth conditions

*V. parahaemolyticus* RMID 2210633 (a clinical strain wild-type strain that harbors both polar and lateral flagella) was cultured at 37 °C in Luria-Bertani (LB) medium supplemented with 1% NaCl. *Escherichia coli* SM10 was cultured at 37 °C with shaking in LB medium. Polar flagella mutant (∆*PFLA*) was created by deleting the ORFs from *vp0773* to *vp0788* (these ORFs encode FlgB, FlgC, FlgD, FlgE FlgG, FlgH, FlgI FlgJ, FlgK, FlgL, FlaC and hypothetical proteins for polar flagella). Lateral flagella mutant (∆*LFLA*) was created by deleting the ORFs from *vpa0264* to *vpa0275* (these ORFs encode LfgB, LfgC, LfgD, LfgE and putative basal body and ring for the lateral flagella). Mutation of *pomA* gene was created by deleting *vp0689*. A suicide vector, pDM4, was used for the creation of the gene deletion mutants as described previously^26^,^47^. Briefly, the upstream and downstream DNA fragment (500 bp) of the genes encoding polar flagella (from *vp0773* to *vp0788*), lateral flagella (*vpa0264* to *vpa0275*) and *pomA* (*vp0689*) was amplified by PCR. Both upstream and downstream DNA fragments were cloned into pDM4. Subsequently, pDM4 harboring the cloned DNA fragments was integrated into the genome of RMID 2210633 by conjugation. After sucrose selection to force the suicide vector to be excised from the genome of *V. parahaemolyticus*, mutants that lack polar flagella, lateral flagella and *pomA* were obtained. Gene deletion mutants were confirmed by sequencing. Complementation was performed by expressing *pomA* in ∆*pomA* strain using a vector, pMMB207, as described previously^26^,^47^.

### Phage isolation and purification

Bacteriophage OWB was isolated from the Atlantic Sea water using the strain RMID 2210633 as described previously (24). The phage propagation procedure was described previously^48^. Initially, phage OWB was propagated using RMID 2210633 as the host strain. Later, we used ATCC17802 *V. parahaemolyticus* strain as the host to propagate phage OWB in order to increase the phage titer because ATCC17802 has very weak polar flagella motility. Briefly, *V. parahaemolyticus* was infected with the phage at a multiplicity of infection (MOI) of 10 and was subsequently incubated at 37 °C for 14 h. Host cell debris was removed by centrifugation at 10,000 rpm for 15 min and subsequent filtration using 0.22 μM filters (Millipore, Billerica, MA). Phages in the filtrate were precipitated in a solution containing 1 M NaCl and 10% polyethylene glycol 8000 (PEG 8000) (final concentration) and incubated on ice for 2 h. After centrifugation at 12,000 rpm at 4 °C for 15 min, the pellet containing the phages was resuspended in sodium chloride magnesium sulfate (SM) buffer (50 mM Tris-HCl, 100 mM NaCl, 10 mM MgSO4 [pH 7.5] [final concentration]). Finally, CsCl density gradient ultracentrifugation was performed at 28,000 rpm at 18 °C for 1 h. The phage particles were collected and dialyzed in a standard dialysis buffer (100 mM MgCl_2_, 10 mM Tris-HCl at pH 7.4). The purified phages were then stored at 4 °C for further experiments.

### Transmission electron microscopy

The morphology of phage OWB was determined by using CsCl-purified phage samples. The samples were negatively stained with 1% uranyl acetate and visualized by electron microscope. To examine phage attachment to bacterial cells, phage OWB was added to the exponentially growing cells of *V. parahaemolyticus* at MOI of 10. After 1 hours of incubation, the mixture of bacteria and phage was negatively stained with 1% uranyl acetate and visualized similarly by electron microscope. To visualize the polar flagella, both WT and ∆*PFLA* were inoculated onto swimming plate (LB containing 0.3% agar) and bacterial cells were stained and process for electron microscopy. Similarly, to visualize the lateral flagella, both WT and ∆*LFLA* were inoculated onto swarm plate (Brain Heart Infusion with 1.5% Bacto agar) and bacterial cells were stained and process for electron microscopy.

### Phage DNA extraction and restriction digestion

Phage DNA was extracted after PEG precipitation. Briefly, pellet of the phage was resuspended in sodium chloride magnesium sulfate (SM) buffer. Protease K (200 μg) and SDS (0.5% final concentration) were added, and the mixture was incubated at 56 °C overnight. Proteins were removed by phenol:chloroform:isoamyl alcohol (25:24:1) extractions, and the nucleic acid was precipitated with alcohol. Finally, the pellets were resuspended in TE buffer (10 mM Tris, pH 8.0, 1 mM EDTA). Restriction enzymes HhaI was used to digest genomic DNA of the phages for 3 h at 37 °C. Electrophoresis of the digested was performed and restriction profile was visualized.

### Bacterial swimming and swarming test

Bacterial swimming was determined on LB media containing 0.3% agar. Overnight culture was spot inoculated swimming plate and swimming ability was recorded after 5 hours of incubation at 37 °C. Bacterial swarming was determined by inoculating the overnight culture onto swarm plate. After overnight incubation at 37 °C, swarming ability was recorded.

### Phage drop assay

To determine the role of polar flagella on phage infectivity, we performed drop assay using WT and Δ*PFLA* strain. Both strains were cultured for 3 hours in LB. Subsequently, bacterial culture (10 μl) of each strain was inoculated on swimming plate as a spot. After the bacterial culture was dried, purified phage OWB (5 μl) was dropped on top of the dried bacterial lawn. After 6 hours of incubation at 37 °C, the clear zone was recorded to reflect the inhibition of bacterial growth. Each experiment was repeated for three times and representative images were shown. To determine the role of lateral flagella on phage infectivity, we performed drop assay using WT and Δ*LFLA* strain. Both strains were cultured for 3 hours in LB. Subsequently, bacterial culture (10 μl) of each strain was inoculated on swarm plate as a spot. Drop assay was performed similarly as described above and the clear zone was recorded after 12 hours of incubation.

### Bacterial growth curve

Fresh *V. parahaemolyticus* culture at OD_600_ of 0.1 was added with phage to reach an MOI of 100. After 1, 2, 3, 4 and 5 hours of shaking at 37 °C, OD_600_ was measured to reflect the bacterial growth. Bacterial growth without addition of phage was also determined for a period of 5 hours. Each experiment was repeated for three times and the average data with standard deviation were presented.

### Phage adsorption assay

Phage at the concentration of 10^7^ pfu/ml was mixed with fresh *V. parahaemolyticus* culture at the concentration of 10^9^ cfu/ml to reach the MOI of 0.01. After incubation at 37 °C for 30 min, the phage-bacteria mixture was centrifuged at 12,000 rpm for 10 min. The free phage titer (pfu/ml) in the supernatant was enumerated and the average data of at least three experiments were presented.

### Phage attachment assay using SYBR green staining

For visualization of phage attachment, both WT and flagella defective strains were transformed with a plasmid pvsv208 that constitutively expresses red fluorescent protein. Phage OWB was labeled with fluorescent dye SYBR green as described previously^49^. Purified phage was mixed with SYBR green and incubated for 15 min at 4 °C in the dark. Subsequently, the mixture was precipitated by PEG/NaCl for 1 h on ice in the dark. After centrifugation at 13,000 rpm at 4 °C for 20 min, the pellet containing the phages was resuspended in SM buffer. The SYBR green-labeled phage was mixed with exponentially growing bacterial cell culture at an MOI of 100. After 1 h of incubation, the mixture of phage and bacteria were visualized using a Zeiss fluorescence microscope. Each experiment was repeated for at least three times and representative images were presented.

### HeLa cytotoxicity assay

Fresh culture of WT and ∆*PFLA* was mixed with phage at an MOI of 100. The mixture was then used to inoculate a 6-well plate containing 10^6^ HeLa cells in each well. After incubation at 37 °C with 5% CO_2_ for about 3 h, HeLa cell supernatant was harvested. Cytotoxicity of *V. parahaemolyticus* against HeLa cells was determined by lactate dehydrogenase (LDH) assay. The supernatant of HeLa cells was collected after 3 hours of inoculation and LDH activity was determined by measuring OD_490_ with a cytotoxicity detection kit (Promega, Madison, WI), according to the manufacturer’s instructions. Bacterial culture without addition of phage was also used to inoculate HeLa cells as a control. Each experiment was repeated for three times and the average data with standard deviation were presented.

### Infection of infant rabbits

Infant rabbits were infected as described previously^27^. Briefly, infant rabbits (2-day old) were orogastrically inoculated with 10^9^ CFU of *V. parahaemolyticus* or both *V. parahaemolyticus* and phage OWB at an MOI of 1. Bacterial colonization (CFU/g intestinal tissue) was measured 38 h post-inoculation. Intestine tissues were fixed in 4% paraformaldehyde and H&E staining was performed for the tissue sections. All animal experiments were performed in accordance with a protocol approved by the IACUC of University of Connecticut (Protocol #A13-060).

### Statistical analysis

Statistical analyses were performed with PRISM software. Comparisons were analyzed using nonparametric one-way analysis of variance (ANOVA) with Bonferroni’s multiple-comparison posttest.

## Additional Information

**How to cite this article**: Zhang, H. *et al.* Polar flagella rotation in *Vibrio parahaemolyticus* confers resistance to bacteriophage infection. *Sci. Rep.* **6**, 26147; doi: 10.1038/srep26147 (2016).

## Figures and Tables

**Table 1:** *V. parahaemolyticus* strains used in this study.

*V. parahaemolyticus*strains	Source	Origin/or genotype	References
RIMD 2210633	clinical	Japan, 1996	25
ATCC17802	clinical	Japan, 1965 ATCC	
∆*PFLA*	RIMD 2210633	Polar flagella knockout	This study
∆*LFLA*	RIMD 2210633	Lateral flagella knockout	This study
∆*pomA*	RIMD 2210633	Vp0689 knockout	This study
∆*pomA*:p*pomA*	RIMD 2210633	Vp0689 knockout	This study
